# Seroprevalence and risk factors of Kaposi's sarcoma-associated herpesvirus infection among the general Uygur population from south and north region of Xinjiang, China

**DOI:** 10.1186/1743-422X-8-539

**Published:** 2011-12-14

**Authors:** Hui Wang, Jian Liu, Dilimulati, Liang Li, Zhihui Ren, Hao Wen, Xing Wang

**Affiliations:** 1 The First Teaching Hospital of Xinjiang Medical University,1 Liyu Shan Road,Urumqi, Xinjiang, People's Taiwan; 2 Institut Pasteur of Shanghai, Chinese Academy of Sciences, Shanghai, People's Taiwan

**Keywords:** Kaposi's sarcoma-associated herpesvirus (KSHV), Kaposi's sarcoma (KS), seroprevalence, Uygur people, risk factors, Xinjiang

## Abstract

**Background:**

Kaposi sarcoma (KS) is a complex multifocal neoplasm and is the major cause of death for about 50% of acquired immunodeficiency syndrome (AIDS) patients. Kaposi's sarcoma-associated herpesvirus (KSHV) is an oncogenic virus with a causal role in the development of all types of KS. KS is prevalent among the Uygur people in Xinjiang, especially in south area. Here we carried out a cross-sectional study among 1534 general Uygur individuals from south and north region of Xinjiang to assess the seroprevalence of KSHV and to identify the potential correlation between KSHV seroprevalence and KS incidence.

**Results:**

Seroprevalence of KSHV in South and North Xinjiang was 23.1% and 25.9%, respectively. Older age was independently associated with higher KSHV seroprevalence. In subjects from South Xinjiang, lower educational level and reported drinking were each independently associated with higher KSHV seroprevalence. Furthermore, the antibody titer was significantly lower in both south and north KSHV seropositive individuals compared with KS patients, as analyzed by gradient dilution (P < 0.001).

**Conclusion:**

KSHV is highly prevalent in the general Uygur population in both South and North Xinjiang. Interestingly, the infection rate of KSHV in these two geographical areas did not correlate well with KS incidence. Perhaps unknown factors exist that promote the progression of KSHV infection to KS development in the local minority groups.

## Background

Kaposi sarcoma (KS) is a mesenchymal tumour involving blood and lymphatic vessels [1]. KS can be classified according to its clinical and epidemiological characteristics and the different types include: classical, acquired immunodeficiency syndrome (AIDS)-related, iatrogenic and endemic KS [2,3]. Notably, KS is the most common cancer associated with AIDS worldwide [4]. Approximately 20% of AIDS patients develop KS in Western countries and AIDS-KS is the major cause of death for about 50% of AIDS patients [5,6]. Kaposi's sarcoma-associated herpesvirus (KSHV) also known as Human herpesvirus 8 (HHV-8 ), is an oncogenic virus with a causal role in the development of KS [2,7-9], and two other AIDS-related lymphoproliferative disorders: primary effusion lymphoma (PEL) and the plasma-cell variant of multicentric Castleman's disease (MCD) [10]. KSHV has been detected in the lesions of nearly all patients with KS [11,12], and when detected in blood it is predictive of the development of KS [8,13].

KSHV prevalence exhibits considerable variation in different geographical regions and populations. Several studies have demonstrated that KSHV seroprevalence correlates with the occurrence of KS [14-17]. In most Asian countries, the seroprevalence of KSHV ranges from 0% to 3%, which is consistent with a generally lower incidence of KS in this region [18]. In most provinces of China, KSHV seroprevalence was less than 8% [19,20]. However, the Xinjiang area, located northwest of China, exhibited a distinct pattern. Over 95% of KS cases in China occurred in Xinjiang, especially classic cases of KS which predominantly occurred in minority groups, particularly in older men [21]. Recent studies have found KSHV seroprevalence correlates with the high incidence of KS in Xinjiang, which ranged from 12.5% to 48.0% in different study populations, including the general population, blood donors, tumor patients and HIV-infected individuals [18,21-24]. The incidence of HIV infection has increased rapidly in Xinjiang over the past few years. Thus, increasing numbers of AIDS-KS cases have recently been reported in this area. It is therefore of great medical importance to investigate KSHV seroprevalence and transmission mode-associated behaviors in Xinjiang, to gain a greater epidemiological understanding of these diseases, and to then be able to apply these findings to improve public health strategies.

Xinjiang is located at the middle point of the Silk Road that used to extend from Rome to China. Many ethnicities, such as the Uygur (48%), Han (38%) and Kazakh people (7%), mix in this area. Classical KS cases occur most frequently in two of these minority groups: the Uygur and Kazakh groups. Furthermore, about 90% of KS cases have been reported in the Uygur group, which reside in the south region of Xinjiang [21]. However, the limitations of previous studies have been that they were generally either restricted to Uygur patients or mainly from the northern part of Xinjiang [18,21,22], where the socio-economic status is higher than in other areas. The objective of the current study was to investigate the potential correlation between KSHV seroprevalence and KS incidence in Xinjiang endemic areas, and to determine whether environmental or sanitation-associated issues effect KSHV infection rates or KS incidence in this ethnic background.

## Results

### Characteristics of the study population and KSHV seroprevalence

Of the 1534 Uygur people included in the study, 1008 (65.7%) were from the southern part of Xinjiang and 526 (34.3%) were from the northern part of Xinjiang. The seroprevalence of KSHV among the general Uygur population in the southern and northern parts of Xinjiang was 23.1% and 25.9%, respectively. Table 1 presents the associations with gender, age and education stratified by region (Southern and Northern). The lower education associated with KSHV seroprevalence only in south region of Xinjiang.

**Table 1:** The associations of KSHV seroprevalence with gender, age and education stratified by geographic region (Southern and Northern) in general Uygur subjects from Xinjiang, China

Characteristic	Moyu country(Southern)				Urumqi(Northern)			
	
	No.of Subjects	KSHV seropositivity n(%)	**χ**^**2**^	*P*	No.of Subjects	KSHV seropositivity n(%)	**χ**^**2**^	*P*
**Gender**			0.337	0.562			0.411	0.521
Male	497	111(22.3)			287	71(24.7)		
Female	511	122(23.8)			239	65(27.2)		
**Age (years)**			2.768	0.096			0.446	0.504
< 40	584	124(21.2)			480	126(26.3)		
≥40	424	109(25.7)			46	10(21.7)		
**Education (years)**			6.632	0.010			2.340	0.126
≤6	720	182(25.3)			24	3(12.5)		
> 6	288	51(17.7)			502	133(26.5)		
**Total**	1088	233(23.1)			526	136(25.9)		

A multivariate logistic regression analysis was used to identify independent risk factors for KSHV infection in total study population (Table 2). In this model, KSHV was significantly more prevalent in individuals aged ≥55 years compared to those aged < 55 years. Moreover, place of residence was also independently associated with KSHV seroprevalence.

**Table 2:** Multivariate logistic regression analysis: determinants of KSHV seropositivity

Characteristic	OR	95% CI	*P *value
**Gender**			
Male	1.00		
Female	1.08	0.86-1.37	0.51
**Age**			
< 40	0.69	0.47-1.03	0.07
40-55	0.63	0.41-0.96	0.03
≥ 55	1.00		
**Education level**			
Illiterate/semiliterate	1.44	0.89-2.30	0.13
Elementary school	1.26	0.90-1.76	0.18
Junior high school	1.00		
**region**			
Moyu country	1.00		
Urumqi	1.49	1.07-2.08	0.02

### KSHV seropositivity among 1008 subjects from south region of Xinjiang

The characteristics of the 1008 Uygur village participants from Southern Xinjiang and the KSHV seroprevalence rates are presented in Table 3. In this study population, 497 subjects were male (49.3%) and 511 were female (50.7%). The age distribution was from 18 to 91 years old. Among 39 subjects positive for hepatitis B and C viruses (HBV and HCV), 11 were KSHV positive (28.2%). Interestingly, participants who were regular drinkers exhibited high serum prevalence of KSHV (46.2%). Table 3 illustrates the univariate associations between KSHV infection and the 1008 participants characteristics. KSHV infection was associated with advancing age(P = 0.020). More specifically, KSHV seroprevalence increased with age from 21.2% in those aged < 40 years to 21.3% in those aged 40-55, and to 30.7% in those aged ≥55 years. Furthermore, there was a significant difference in KSHV seroprevalence with respect to educational level (P = 0.020), from 17.7% in those educated to junior high school level, to 24.3% in those educated to elementary school level, and to 28.3% in those who were illiterate/semiliterate. An association of borderline significance was detected between KSHV seropositivity and the report of drinking (P = 0.058). No associations were observed between KSHV infection and gender, family number, BMI and HBV or HCV infection.

**Table 3:** Univariate analysis of KSHV seroprevalence in the subjects from southern part of Xinjiang

Characteristic	Uygur people n(%)	KSHV seropositivity n(%)	OR	95% CI	*P *value
**Gender**					
Male	497	111 (22.3)	1.00		
Female	511	122 (23.8)	1.10	0.81-1.46	0.56
**Age**					
< 40	584	124 (21.2)	1.00		
40-55	225	48 (21.3)	1.01	0.69-1.47	0.98
≥55	199	61 (30.7)	1.64	1.14-2.35	0.01
**Education level**					
Illiterate/semiliterate	180	51 (28.3)	1.00		
Elementary school	540	131 (24.3)	0.81	0.55-1.18	0.28
Junior high school	288	51 (17.7)	0.54	0.35-0.85	0.01
**Family number**					
≤5 people	641	151 (23.6)	1.00		
> 5 people	367	82 (22.3)	0.93	0.69-1.27	0.66
**Smoking**					
No	931	217 (23.3)	1.00		
Yes	77	16 (20.8)	0.86	0.49-1.53	0.61
**Drinking**					
No	995	227 (22.8)	1.00		
Yes	13	6 (46.2)	2.90	0.97-8.72	0.06
**HBV**					
Negative	983	226 (23.0)	1.00		
Positive	25	7 (28.0)	1.30	0.54-3.16	0.56
**HCV**					
Negative	994	229 (23.0)	1.00		
Positive	14	4 (28.6)	1.34	0.42-4.30	0.63
**BMI**					
≥25	330	73 (22.1)	1.00		
< 25	678	160 (23.6)	0.92	0.67-1.26	0.60
**Total**	1008	233 (23.1)			

To further identify independent risk factors, variables that were significant in univariate analysis at P = 0.100 were included in multivariate logistic regression analysis and the data are presented in Table 4. Lower educational level (Illiterate/semiliterate vs. Junior high school, OR = 1.67, 95% CI = 1.02-2.73) and reported drinking (yes vs. no, OR = 3.23, 95%CI = 1.07-9.78)were each independently associated with higher KSHV seroprevalence.

**Table 4:** Independent risk factors for KSHV infection in southern part of Xinjiang

Characteristic	OR	95% CI	*P *value
**Age (years)**			
< 40	1.00		
40-55	0.91	0.62-1.35	0.64
≥55	1.42	0.95-2.11	0.08
**Education level**			
Junior high school	1.00		
Elementary school	1.48	1.02-2.14	0.04
Illiterate/semiliterate	1.67	1.02-2.73	0.04
**Drinking**			
No	1.00		
Yes	3.23	1.07-9.78	0.04

### Antibody titers of KSHV in highly seropositive individuals and KS patients

**Figure 1:**
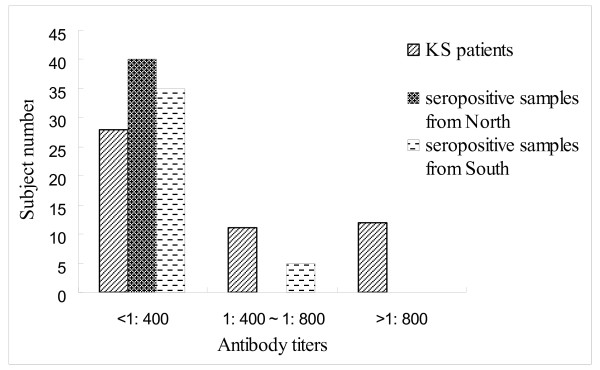
**Distribution of KSHV antibody titers in KS patients and seropositive samples from North and South Xinjiang**.

The distribution pattern of KSHV antibody titers among high seropositive individuals from both north and south and KS patients were compared and the results are displayed in Figure 1. The individual number of low antibody titer group in south, north and KS patients was 35,40 and 28. For medium group, the number is 5,0 and 11. However, the number is 0,0 and 12 in south, north and KS patients for high group( χ^2 ^= 33.74, P < 0.001).These results indicated that compared with KS patients, KSHV-infected individuals in both south and north presented a lower antibody titer against KSHV.

## Discussion

More than 95% of KS cases in China, including all epidemiological forms, were observed in minority groups in Xinjiang, particularly in the Uygur group [21]. Previous studies have shown a high seroprevalence of KSHV in the range of 12.5% to 48.0% in different study populations in this region, that correlated well with a high incidence of KS [18,21-23]. However, these studies were mainly restricted to Uygur patients with diseases other than KS, and the study populations were mainly from the northern part of Xinjiang [18,21]. Thus the discrepancy between the socio-economic status in these two geographical areas and the absence of behavior-associated data, mean that these studies did not take other variables into account. In the current cross-sectional study, the seroprevalence of KSHV among the general Uygur population in the southern and northern parts of Xinjiang was 23.1% and 25.9%, respectively, which is in agreement with previous findings from Xinjiang [21-24]. There was no significant difference in KSHV infection between the two regions of Xinjiang, indicating that minority group background is still the most important factor in KSHV infection. Although as expected, titers were higher in KS cases than in the general population, titers in the general population did not differ by geography, unlike the incidence of KS which is much higher in the South. Combined with the distribution pattern of KSHV antibody titers among seropositive individuals and KS patients, our findings suggested that other unknown factors may exist in South Xinjiang which potentially promote the development from KSHV infecton during KS pathogenesis. To further confirm this hypothesis, a prospective study focused on geographical and ethnic parameters should be carried out.

The seroprevalence of KSHV in the general Uygur population in Xinjiang was significantly more prevalent in individuals aged ≥55 years compared to those aged < 55 years. These results are consistent with those of several other studies, which described an increase in seroprevalence of KSHV with age in different populations [25-29]. This correlation may be related to the natural aging process. Generally, the immunity of older people is weaker than that of younger people, and hypoimmunity maybe a promoting agent for KSHV infection. Moreover, it is notable that KSHV prevalence was similar in men and women which is consistent with many studies [18,29]. and our previous researches [21,23] that find the distribution of KSHV seroprevalence has no difference by sex.

Further analyses were then carried out to focus on the 1008 rural Uygur people from South Xinjiang. This is the first report regarding KSHV seroprevalence and risk factors in this area, a region endemic for KS. The seroprevalence of KSHV in this study population was independently associated with educational level. It was apparent that with improved educational level (from illiterate/semiliterate to Junior high school level education), KSHV seroprevalence decreased from 28.3% to 17.7%, suggesting that a higher education level is a protection factor for KSHV infection. Notably, the educational level of this study population was usually below the high school level, and among the older age group (≥55) the percentage of illiterate/semiliterate individuals was 43.7%. Therefore, knowledge about health issues is far more limited in the Uygur population in this region, especially in the elder Uygur population. Furthermore, the mobility of the population in this region is relatively low. The traditional life style and behavior of the Uygur people has remained unchanged over the past two decades [30], with the life quality and level of education reflecting their low socioeconomic status. Such conditions might facilitate the transmission of KSHV infection, in line with with previous reports linking KSHV to rural settings in Italy and Sicily [31-33]. Interestingly, we also identified drinking could increase the risk of KSHV infection, a result consistent with previous studies [34,35]. Among 13 drinkers, six were KSHV positive (46.2%). Because of the small sample size, the relationship between drinking and KSHV infection requires further investigation in a larger sample population in Xinjiang.

## Conclusions

We identified a high seroprevalence of KSHV infection in the general Uygur population in both South and North Xinjiang, and the distribution of KSHV seroprevalence in these two geographical areas did not show a clear correlation with KS incidence. Perhaps other unknown factors have promoted the association between KSHV infection and KS development in the local minority groups. Understanding the epidemiology of KSHV infection in rural areas of Uygur is critical for designing effective intervention strategies to decrease the transmission of this virus and to prevent malignancies associated with KSHV infection.

## Methods

### Study participants

South Xinjiang was represented by Moyu Country of Hotan City which is located in the southernmost region of Xinjiang surrounding the Tarim Basin and where more than 95% of the population were rural Uygur people. North Xinjiang was represented by Urumqi, which is the most highly developed and industrialized region in Xinjiang. A total of 1543 research subjects were randomly selected from this two regions of Xinjiang by using a stratified, multistage sampling. Among them, residents (age > 18years) from 15 villages in 3 rural town of Moyu Country and employees from top 10 developed enterprises of Urumqi were 1008 and 526, respectively. Trained staff obtained the informed consent of all participants and carried out interviews. A questionnaire on age, gender, ethnicity, education level and residence was completed for all subjects. Other characteristics, such as family number, drinking, cigarette smoking and anthropometric measurements, including height, weight, waistline and hip circumference, were also recorded for all of the subjects in the southern part of Xinjiang. All data were analyzed anonymously. Body mass index (BMI) (kg/m^2^) values were also calculated for all subjects. Serum and plasma samples were collected, separated immediately and stored at -80°C prior to testing.

Sera were also collected from samples taken from 51 patients with classic KS registered during the period between 1986 and 2008, at the First Teaching Hospital of Xinjiang Medical University in Urumqi (age: 17-83 years, 45 male vs. 6 female, 46 Uygur vs. 5 Kazakh, 46 from south region vs. 5 from other region of xinjiang), to compare antibody titers with KSHV infected persons.The study protocol was approved by the local research ethics committee of the First Teaching Hospital of Xinjiang Medical University. Written informed consents were obtained from all study subjects before data collection.

### Serological tests for KSHV

Three KSHV specific viral genes were expressed and purified as GST-fusion proteins used to construct the combined antigens enzyme-linked immunosorbent assay(ELISA) including latent antigen ORF73 and lytic antigens ORF K8.1 and ORF65. Briefly, Viral antigens were diluted in coating buffer to a final concentration of 10 μg/ml and used to coat ELISA plates (Corning Glass Works, Corning, NY, USA) overnight at 4°C. After washing with phosphate-buffered saline (PBS) containing 0.05% Tween 20(PBST), 1:100 diluted serum samples were added and plates were incubated for 60 min at 37°C. This was followed by incubation with goat anti-human IgG conjugated with peroxidase (Product No. A8667, Sigma, St. Louis, MO, USA) for 60 min at 37°C. The color reaction was developed for 15 min at RT with tetramethyl benzidine after PBST washing. Reactions were stopped with 50 μl of 1 M H_2_SO_4 _and absorbance was measured at a wavelength of 450 nm. Serum samples from patients with classical-KS in Xinjiang and with AIDS-KS or skin carcinoma in France which were obtained from the Medical center of Besancon University France-Comte were used to assay the sensitivity and specificity in this study. Based on the surveys of above groups, the combined antigens ELISA had a sensitivity of 81.8% and specificity of 97.9%, respectively. In the following screening, serum from an AIDS-KS patient that had high antibody titers to both KSHV latent and lytic antigens, and serum from three healthy children in Sichuan province without any specific antibodies to KSHV, were used as positive and negative controls, respectively, in all assays. Both positive and negative controls were used in three wells in each plate in this study. Each sample was also tested three times. Based on the results of assays with the control groups, a serum sample with an absorbance value above the mean plus three standard deviations of the negative control wells in an assay was considered as positive. A highly seropositive result for KSHV was set considered as a value greater than the mean absorbance value plus five standard deviations of the negative control wells. All assays were examined by a single observer.

Serum samples that were highly positive for antibodies against KSHV in the ELISA assay and the sera of 51 KS patients were analyzed to determine their antibodiy titers using gradient serum dilutions (1:100,1:200,1:400,1:800, 1:1600 and 1:3200). The positive standard followed the criterion above. Antibody titers lower than 1:400, between 1:400~1:800 and higher than 1:800 were classified into the low group, medium and high group, respectively.

### Statistical analysis

The data were entered in duplicate and analyzed using the SPSS11.0 software (SPSS, Chicago, IL).Univariate analysis was performed to examine associations between the KSHV serostatus and all of the questionnaire variables. Factors that were significant in univariate analyses at P = 0.100 were included in a multivariate logistic regression analysis, to identify which factors, if any, were independently associated with KSHV infection. Odds ratios (ORs) and 95% confidence intervals (CIs) were used to quantify the relationships, while P-values were calculated to indicate the statistical significance. P-values < 0.050 were considered significant. The chi-square test was also performed when appropriate to compare antibody titers between KSHV -infected individuals and KS patients.

## List of abbreviations

KS: Kaposi sarcoma; KSHV: Kaposi's sarcoma-associated herpesvirus; HHV-8: Human herpesvirus 8; AIDS: acquired immunodeficiency syndrome; PEL : primary effusion lymphoma; MCD: multicentric Castleman's disease; OR: Odds ratios; 95%CI: 95% confidence intervals; HBV: hepatitis B viruses; HCV: hepatitis C viruses; BMI: Body mass index; ELISA: enzyme-linked immunosorbent assay; RT: room temperature; ORF: open reading frame

## Competing interests

The authors declare that they have no competing interests.

## Authors' contributions

HW carried out study design, sample collection, and statistical analyses performance and participated in the antibody detection. JL and LL participated in sample collection. Dilimulati and ZH R wrote and collected the questionnaire. HW participated in the design of the study and carried out statistical analyses. XW conceived of the study, and participated in its design and coordination and helped to draft the manuscript. All authors read and approved the final manuscript.

## References

[B1] Simonart Thierry Role of environmental factors in the pathogenesis of classic and African-endemic Kaposi sarcoma Cancer Letters 2006 244 1 7 10.1016/j.canlet.2006.02.005 16542773

[B2] Chang Y Cesarman E Pessin MS Identification of herpesvirus-like DNA sequences in AIDS-associated Kaposi's sarcoma Science 1994 266 1865 1869 10.1126/science.7997879 7997879

[B3] Schalling M Ekman M Kaaya EE A role for a new herpesvirus (KSHV) in different forms of Kaposi's sarcoma Nat Med 1995 1 707 708 10.1038/nm0795-707 7585156

[B4] Hladik W Dollard SC Mermin J Transmission of human herpesvirus 8 by blood transfusion N Engl J Med 2006 355 1331 1338 10.1056/NEJMoa055009 17005950

[B5] Beral V Peterman TA Berkelman RL Kaposi's sarcoma among persons with AIDS: a sexually transmitted infection? Lancet 1990 335 123 128 10.1016/0140-6736(90)90001-L 1967430

[B6] Giovanni S Antonio S Barbara I Sirolimus for Kaposi's Sarcoma in Renal-Transplant Recipients N Engl J Med 2005 352 1317 1323 10.1056/NEJMoa042831 15800227

[B7] Gao SJ Kingsley L Li M KSHV antibodies among Americans, Italians and Ugandans with and without Kaposi's sarcoma Nat Med 1996 2 925 928 10.1038/nm0896-925 8705864

[B8] Whitby D Howard MR Tenant-Flowers M Detection of Kaposi's sarcoma associated herpesvirus in peripheral blood of HIV-infected individuals and progression to Kaposi's sarcoma Lancet 1995 346 799 802 10.1016/S0140-6736(95)91619-9 7674745

[B9] Zavitsanou A Malloiri M Sypsa V Seroepidemiology of human herpesvirus 8 (HHV-8) infection in injecting drug users Epidemiol Infect 2010 138 403 408 10.1017/S0950268809990628 19698211

[B10] Abel VB Thomas FS. Kaposi's Sarcoma-associated Herpesvirus(KSHV/HHV8): Key Aspects of Epidemiology and Pathogenesis AIDS Rev 2003 5 222 229 15012001

[B11] Boshoff C Schulz TF Kennedy MM Kaposi's sarcoma-associated herpesvirus infects endothelial and spindle cells Nat Med 1995 1 1274 1278 10.1038/nm1295-1274 7489408

[B12] Staskus KA Zhong W Gebhard K Kaposi's sarcoma-associated herpesvirus gene expression in endothelial (spindle) tumor cells J Virol 1997 71 715 719 8985403 10.1128/jvi.71.1.715-719.1997 PMC191104

[B13] Moore PS Kingsley LA Holmberg SD Kaposi's sarcoma-associated herpesviurs infection prior to onset of Kaposi's sarcoma AIDS 1996 10 175 180 10.1097/00002030-199602000-00007 8838705

[B14] Kedes DH Ganem D Ameli N The prevalence of serum antibody to human herpesvirus 8 (Kaposi sarcoma-associated herpesvirus) among HIV-seropositive and high-risk HIV-seronegative women JAMA 1997 277 478 481 10.1001/jama.1997.03540300046032 9020272

[B15] Rezza G Lennette ET Giuliani M Prevalence and determinants of anti-lytic and anti-latent antibodies to human herpesvirus-8 among Italian individuals at risk of sexually and parenterally transmitted infections Intl J Cancer 1998 77 361 365 10.1002/(SICI)1097-0215(19980729)77:3<361::AID-IJC9>3.0.CO;2-M 9663596

[B16] Gao SJ Kingsley L Li M KSHV antibodies among Americans, Italians and Ugandans with and without Kaposi's sarcoma Nat Med 1996 2 925 928 10.1038/nm0896-925 8705864

[B17] Boshoff C Weiss RA Epidemiology and pathogenesis of Kaposi's sarcoma-associated herpesvirus Phil Trans R Soc Lon Biol Sci 2001 356 517 534 10.1098/rstb.2000.0778 PMC1088442 11313009

[B18] Fu B Sun F Li B Seroprevalence of Kaposi's Sarcoma-associated Herpesvirus and Risk Factors in Xinjiang, China J Med Virol 2009 81 1422 1431 10.1002/jmv.21550 19551832 PMC2755560

[B19] Fang Q Liu J Q BZ Seroprevalence of Kapsosi's sarcom-associated herpesvirus in the central population from Hubei provine Viroligica Sinica 2006 21 97 101

[B20] Wang GQ Xu H Wang YK Higher prevalence of human herpesvirus 8 DNA sequence and specific IgG antibodies in patients with pemphigus in China J Am Acad Dermatol 2005 52 460 467 10.1016/j.jaad.2004.10.882 15761424

[B21] He F Wang X He B Human herpesvirus 8: serovprevalence and correlates in tumor patients from Xinjiang, China J Med Virol 2007 79 161 166 10.1002/jmv.20730 17177299

[B22] Du W Chen G Sun H Antibody to human herpesvirus type 8 in the general populations of Xinjiang Autonomous Region Chinese J Exp ClinVirol 2000 14 44 47 11503024

[B23] Wang X He B Zhang ZX Human herpesvirus-8 in northwestern China: epidemiology and characterization among blood donors Virology Journal 2010 7 62 69 10.1186/1743-422X-7-62 20236530 PMC2852390

[B24] Sitas F Carrara H Beral V Antibodies against human herpesvirus 8 in black South African patients with cancer N Engl J Med 1999 340 1863 1871 10.1056/NEJM199906173402403 10369849

[B25] Baillargeon J Deng JH Hettler E Seroprevalence of Kaposi's sarcoma-associated herpesvirus infection among blood donors from Texas Ann Epidemiol 2001 11 512 518 10.1016/S1047-2797(01)00242-3 11557184

[B26] Sitas F Newton R Kaposi's sarcoma in South Africa J Nati Cancer Inst Monogr 2001 28 1 4 10.1093/oxfordjournals.jncimonographs.a024250 11158199

[B27] Stein L Carrara H Norman R Antibodies against human herpesvirus 8 in South African renal transplant recipients and blood donors Transplant Infection Disease 2004 6 69 73 10.1111/j.1399-3062.2004.00061.x 15522107

[B28] Zavitsanou A Sypsa V Petrodaskalaki M Human herpesvirus 8 (HHV-8) infection in healthy urban employees from Greece: Seroprevalence and associated factors J Med Virol 2007 79 591 596 10.1002/jmv.20812 17385692

[B29] Zavitsanou A Malliori M Sypsa V Seroepidemiology of human herpesvirus 8 (HHV-8) infection in injecting drug users Epidemiol Infect 2010 138 403 408 10.1017/S0950268809990628 19698211

[B30] Li N GUO SX Zhang YH Investigation on knowledge, attitude and behaviour regarding hypertension among the Kazakh residents in Xinjiang Journal of Shihezi University 2007 25 203 206

[B31] Angeloni A Masala MV Montesu MA Environmental factors influence the rate of human herpesvirus type 8 infection in a population with high incidence of classic Kaposi sarcoma Clin Infect Dis 2006 42 e66 e68 10.1086/500397 16511749

[B32] Valdarchi C Serraino D Cordiali Fei P Demographic indicators and risk of infection with human herpesvirus type 8 in central Italy Infection 2007 35 22 25 10.1007/s15010-007-5123-2 17297585

[B33] Pelser C Vitale F Whitby D Socio-Economic and Other Correlates of Kaposi Sarcoma-Associated Herpesvirus Seroprevalence Among Older Adults in Sicily J Med Virol 2009 81 1938 1944 10.1002/jmv.21589 19777527 PMC2784645

[B34] Wojcicki JM Newton R Urban MI Risk factors for high anti-HHV-8 antibody titers (≥1:51 200)in black HIV-1 negative South African cancer patients: a case control study BMC Infect Dis 2003 3 21 32 10.1186/1471-2334-3-21 12971827 PMC222909

[B35] Mbulaiteye S Atkinson J Whitby D Risk factors for human herpesvirus 8 seropositivity in the AIDS Cancer Cohort Study J Clin Virol 2006 35 442 449 10.1016/j.jcv.2005.10.010 16414306

